# Modelling Climate and Societal Resilience in the Eastern Mediterranean in the Last Millennium

**DOI:** 10.1007/s10745-018-9995-9

**Published:** 2018-04-19

**Authors:** Elena Xoplaki, Jürg Luterbacher, Sebastian Wagner, Eduardo Zorita, Dominik Fleitmann, Johannes Preiser-Kapeller, Abigail M. Sargent, Sam White, Andrea Toreti, John F. Haldon, Lee Mordechai, Deniz Bozkurt, Sena Akçer-Ön, Adam Izdebski

**Affiliations:** 1 Climatology, Climate Dynamics and Climate Change, Department of Geography, Justus-Liebig-University Giessen, Giessen, Germany; 2 Centre of International Development and Environmental Research, Justus-Liebig-University Giessen, Giessen, Germany; 3 Institute for Coastal Research, Helmholtz-Zentrum Geesthacht, Geesthacht, Germany; 4 Department of Archaeology and Centre for Past Climate Change, School of Human and Environmental Sciences, University of Reading, Reading, UK; 5 Institute for Medieval Research/Division of Byzantine Research, Austrian Academy of Sciences, Vienna, Austria; 6 History Department, Princeton University, Princeton, NJ USA; 7 History Department, Ohio State University, Columbus, OH USA; 8 European Commission, Joint Research Centre, Ispra, Italy; 9 Center for Climate and Resilience Research, Department of Geophysics, University of Chile, Santiago, Chile; 10 Geological Engineering Department, Mugla Sitki Kocman University, Mugla, Turkey; 11 Byzantine History Department, Institute of History, Jagiellonian University in Krakow, Krakow, Poland; 12 School of Historical Studies, Institute for Advanced Study, Princeton, NJ USA; 13 Max Planck Institute for the Science of Human History, Jena, Germany

**Keywords:** Resilience, Eastern Mediterranean, Palaeoclimatology, CMIP5 models, Model/data comparison, First millennium, Middle Ages, Early modern period, Complex societies

## Abstract

This article analyses high-quality hydroclimate proxy records and spatial reconstructions from the Central and Eastern Mediterranean and compares them with two Earth System Model simulations (CCSM4, MPI-ESM-P) for the Crusader period in the Levant (1095–1290 CE), the Mamluk regime in Transjordan (1260–1516 CE) and the Ottoman crisis and Celâlî Rebellion (1580–1610 CE). During the three time intervals, environmental and climatic stress tested the resilience of complex societies. We find that the multidecadal precipitation and drought variations in the Central and Eastern Mediterranean cannot be explained by external forcings (solar variations, tropical volcanism); rather they were driven by internal climate dynamics. Our research emphasises the challenges, opportunities and limitations of linking proxy records, palaeoreconstructions and model simulations to better understand how climate can affect human history.

## Introduction

The interaction between environmental stress and socio-political systems is increasingly attracting the interest of the scientific community and the general public (e.g., Büntgen *et al*. 2011, 2016; White 2011; McCormick *et al*. 2012; Haldon *et al*. 2014; Luterbacher and Pfister 2015; Preiser-Kapeller 2015; Xoplaki *et al*. 2016; Fuks *et al*. 2017; Manning *et al*. 2017; Haldon and Rosen 2018; Izdebski A, Mordechai L, and White S. The costs of resilience in premodern societies. Human Ecology, under review). Although understanding how past societies responded to extreme climatic changes is crucial for gaining insight into current and future environmental challenges (e.g., Luterbacher and Pfister 2015; Haldon 2016; Haldon *et al*. 2018), very few scientific studies have comprehensively addressed the multi-faceted character of socio-cultural resilience (Haldon *et al*. 2018; Izdebski A, Mordechai L, and White S. The costs of resilience in premodern societies. Human Ecology, under review). This is not surprising, given the challenges entailed in this type of interdisciplinary approach.

Haldon and Rosen (2018) highlight methodological issues regarding scales, data analysis and interpretation, the compatibility of different types of data (social scientific and natural scientific), and the predictive value of modelling these relationships for understanding past societal and cultural change and re-assessing interpretations of these different types of data. In particular, scholars must be wary of simplistic explanations of the role of climate in human history (environmental determinism), and every observation or conclusion should remain provisional (Izdebski *et al*. 2016).

To study the complex interactions between climate and past societies we need a dense network of reliable palaeoclimate records of high temporal resolution, dating precision, and resolved climate information, as well as appropriate types of historical data. Compared with most parts of the world, the Mediterranean region has a wealth of natural climate archives and historical and archaeological data that make the area a perfect natural laboratory to reveal such potential linkages (e.g., Luterbacher *et al*. 2012; McCormick *et al*. 2012; Haldon *et al*. 2014; Izdebski *et al*. 2016; Sadori *et al*. 2016; Weiberg *et al*. 2016; Labuhn *et al*. 2018; Roberts *et al*. 2018).

Palaeoevidence clearly indicates that climatic fluctuations in the Mediterranean over the past millennium have not been homogeneous but have demonstrated a high degree of variability over time and across space (e.g., Luterbacher *et al*. 2012; Roberts *et al*. 2012; Cook *et al*. 2016; Labuhn *et al*. 2018; Luterbacher and Xoplaki 2018; Roberts *et al*. 2018). Hydrological changes in particular are more challenging for societies in the Mediterranean area than temperature fluctuations since precipitation variability can impact agriculture and food production. The Eastern Mediterranean is particularly drought-prone and water stressed, which can have strong direct impacts on livestock and crops, and indirect impacts related to nutrition, loss of livelihood, sanitation, displaced populations, and international conflicts (Barlow *et al*. 2016).

We discuss possibilities, challenges, limitations and uncertainties of the palaeoclimate sources, the historical-archaeological evidence, reconstructions and how they can be integrated and compared with climate model simulations to better understand resilience of complex societies of the past. We use three case studies: the Crusader Levant (1095–1290 CE), the Mamluk regime in Transjordan (1260–1516 CE), and the Ottoman crisis and Celâlî Rebellion (1580–1610 CE). We offer new insights into the climatic conditions of those selected periods through interpretation of hydro-climate variations and trends from various proxy records, and analysis of spatial reconstructions of temperature (Luterbacher *et al*. 2004; Xoplaki *et al*. 2005), precipitation (Pauling *et al*. 2006), and drought (Cook *et al*. 2015) in the context of the past millennium. We compare the climatic evidence from the Central and Eastern Mediterranean with two state-of-the-art model simulations (CCSM4 and MPI-ESM-P) from the Coupled Model Intercomparison Project Phase 5 (CMIP5, Taylor *et al*. 2012). We highlight important processes and underlying dynamics during the three periods when complex societies underwent significant economic or political transformations associated with environmental stresses and corresponding adaptations.

## Climate

### Seasonal Large-Scale Temperature, Precipitation and Drought Reconstructions

**Fig. 1:**
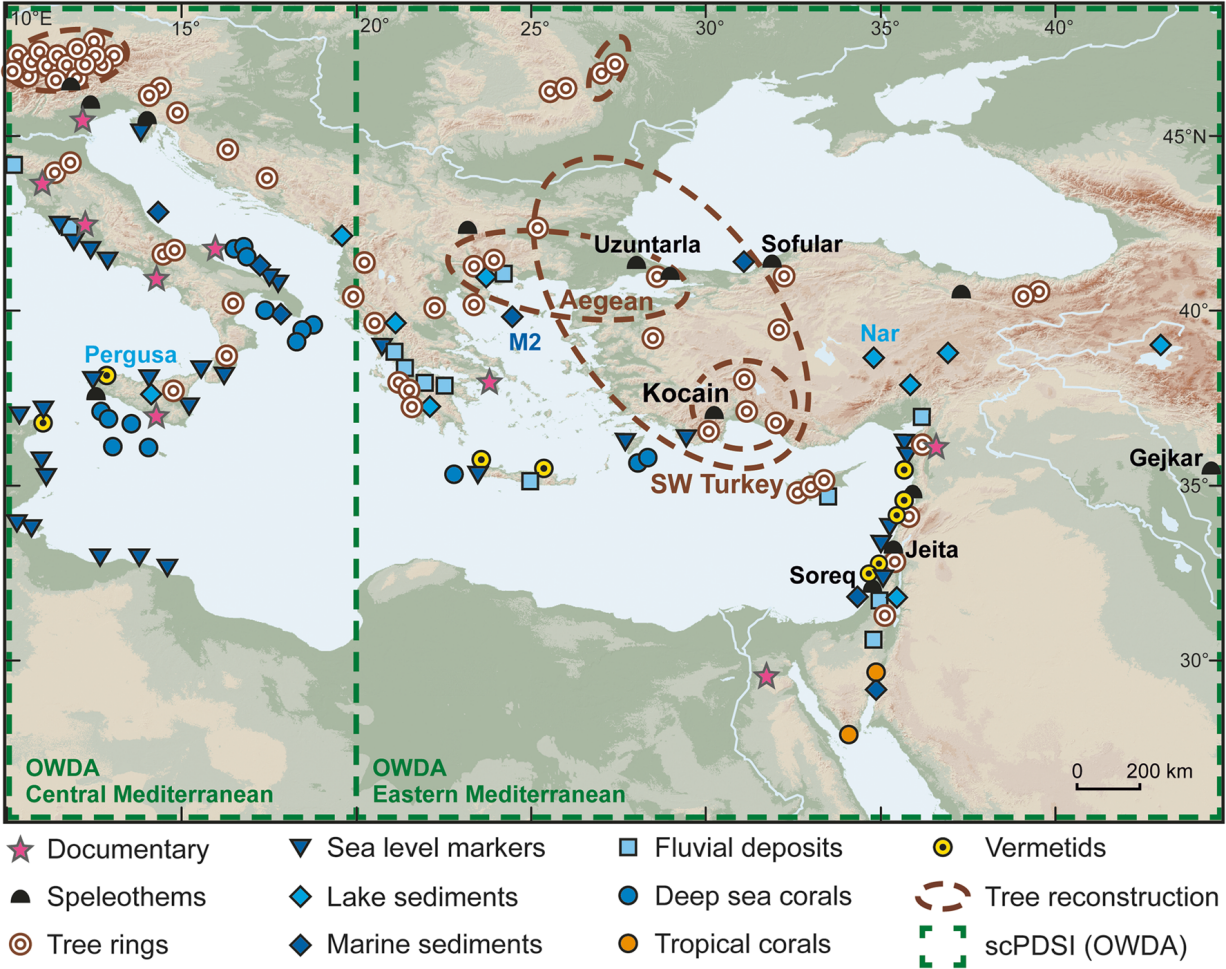
Location of terrestrial and marine proxies available in the Central and Eastern Mediterranean that cover at least 600 years of information. The proxy records vary in length and the proxies provide different climate information and record climate conditions during different seasons. The names of the proxy records used in this paper are given on the map together with the Old World Drought Atlas (OWDA; Cook *et al*. 2015) domains for the Central and Eastern Mediterranean (see Fig. 2 for the proxy records time series covering the past millennium)

Historical instrumental weather observations from the sea and land as early as the seventeenth century are integral to extending our understanding of past climates and for comparison with palaeoproxy data (Camuffo *et al*. 2010a, b). Modern meteorology began in Italy, where Galileo (1564–1642) claimed to have invented the first thermometer. His students were involved in the first concerted efforts to develop meteorological instrumentation for gathering detailed weather information (Camuffo *et al*. 2010a, b). Widespread meteorological instrumental observations from the Mediterranean are available for approximately the last 70 years and up to two centuries at a few specific locations (Fig. 1 in Luterbacher *et al*. 2012). Indirect climate indicators from natural archives and documentary evidence are used to characterize past climate conditions and variations for the pre-instrumental period (see Luterbacher *et al*. 2012 for an overview of the Mediterranean) (Fig. 1).

The proxy records are sensitive to multiple climate signals (e.g., precipitation, temperature, sea level changes, sea surface temperature, water circulation, and pH), and the lengths of their time series vary. Their temporal resolution ranges from seasonal (such as tree rings) to multi-decadal (such as marine deep-sea corals and marine sediments, e.g., Gogou *et al*. 2016) and they represent climate conditions during different parts of the year (Luterbacher *et al*. 2012). Sensitivity, reproducibility, local availability and continuity throughout time periods may also differ (Mann 2002). Proxy-based reconstructions include uncertainties associated with using imperfect proxy and target (instrumental) data and the uncertainty associated with the applied statistical methodology. Data uncertainties include measurement errors in the proxies, uncertainty in proxy-temperature/precipitation relationships (which can be contaminated by the effect of other variables), sampling errors in instrumental data, chronological uncertainties, and uncertainty resulting from the network’s coarse spatio-temporal coverage. Methodological uncertainties include the sensitivity to model parameters and the sensitivity of the method related to its input data (resolution, type of data, noise level, and spatio-temporal variability).

### Hydroclimate Proxy Evidence from the Central and Eastern Mediterranean

**Fig. 2:**
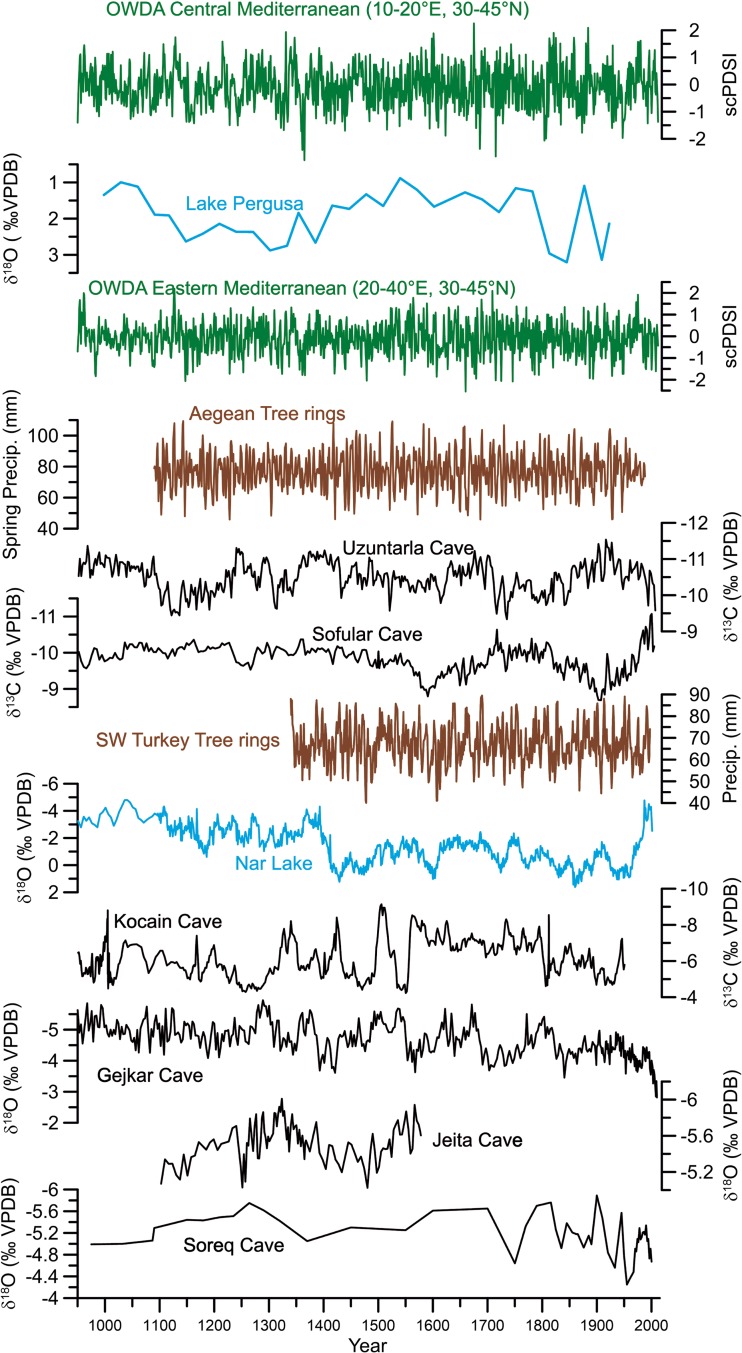
Hydroclimate proxy evidence from Central and Eastern Mediterranean for the past millennium (see Fig. 1 for corresponding locations)

Although the number of palaeoclimate reconstructions for most areas of the Middle East has increased during the last years, records are subject to seasonal biases (e.g., tree rings), chronological uncertainties (e.g., lake sediments, speleothems), and/or low temporal resolution (e.g., marine and lake sediments). Furthermore, there are still spatial gaps in many areas of the Middle East, Turkey, and Northern Africa in particular. These limitations make it difficult to correlate variations in hydrological changes with regional to basin-wide socio-economic transformations and historical events. High-quality palaeoclimate records for parts of the Eastern Mediterranean and Middle East reflect parts of the hydrological cycle including changes in precipitation and effective moisture, respectively (Fig. 2).

Regional tree ring records from the Aegean (Griggs *et al*. 2007) and Southwest Turkey (Touchan *et al*. 2003) are closely related to spring precipitation that accounts for less than 20% of total annual rainfall amount (Xoplaki *et al*. 2004). Both tree ring reconstructions show distinct fluctuations in spring precipitation, whereas decadal and multi-decadal fluctuations are not apparent, a known limitation of tree ring-based reconstructions. Both tree ring chronologies are part of the Old World Drought Atlas (OWDA, Cook *et al*. 2015), which provides tree ring-based field reconstructions of summer season self-calibrating Palmer Drought Severity Index (scPDSI). The drought atlas is spatially resolved to 0.5°×0.5° and covers only land areas. The longest available tree ring chronologies are well distributed across the Mediterranean region, with the greatest densities in Anatolia, the Western Mediterranean, and Northern Italy (Cook *et al*. 2015).

Multi-decadal fluctuations can be found in oxygen (δ^18^O) and carbon (δ^13^C) isotope records obtained from stalagmites and lake sediments across the Middle East. The Pergusa Lake (Sicily; Sadori *et al*. 2016) and Nar Lake (Central Anatolia; Jones *et al*. 2006) δ^18^O records are strongly governed by the precipitation-evaporation balance and therefore provide indirect information on the amount of precipitation. The stalagmite δ^18^O records from the caves at Uzuntarla (Göktürk 2011), Sofular (Göktürk *et al*. 2011), Gejkar (Flohr *et al*. 2017), Jeita (Cheng *et al*. 2015), and Soreq (Bar-Matthews *et al*. 2003) also show both long-term and decadal to multi-decadal variations in effective moisture. Variations in effective moisture are not coherent among the stalagmite records, a result that is mainly related to the strong regional climatic differences among the cave sites in Turkey, the Levant, and Iraqi Kurdistan. Almost all of the stalagmite δ^18^O and δ^13^C records carry chronological uncertainties ranging from a few years (e.g., Gejkar Cave; Flohr *et al*. 2017) up to several decades (e.g., Uzuntarla, Kocain, and Soreq; Göktürk 2011; Bar-Matthews *et al*. 2003). This increases the complexity of associating rather short historical events such as the Celâlî Rebellion with climatic events in the stalagmite records. Interestingly, there is no evidence in any of the stalagmite records (Figs. 1 and 2) for a typical Medieval Climate Anomaly (MCA)-Little Ice Age (LIA) pattern, but all oxygen isotope time series are marked by distinct decadal to multi-decadal changes in effective moisture.

Below, we compare these hydro-climatic proxies and derived precipitation/moisture proxies with different temporal resolution from various regions across the Central and Eastern Mediterranean (Figs. 1 and 2) with the CCSM4 and MPI-ESM-P simulations for the periods 1220–1250 CE, 1260–1450 CE and 1580–1610 CE.

In addition, we use spatial gridded temperature (Luterbacher *et al*. 2004; Xoplaki *et al*. 2005), precipitation (Pauling *et al*. 2006), and drought (Cook *et al*. 2015) reconstructions covering the past 500 years for the comparison with model simulations during the Celâlî Rebellion (1580–1610 CE). The reconstructions provide spatially highly resolved, seasonal data based on a multivariate calibration of proxies across Europe and the Mediterranean against twentieth-century gridded (with 0.5° × 0.5° spatial resolution) land-based meteorological data. The statistical relationships between the proxies and the instrumental data were applied to the pre-instrumental period using the available proxies, resulting in seasonal precipitation/temperature and drought patterns back to 1500 CE.

### Climate Model Simulations

Comprehensive Earth System Models (ESMs) simulate the atmospheric and oceanic system, land use changes including the terrestrial carbon cycle, atmospheric chemistry, ocean biochemistry, and the interactions among these systems. The high complexity ESMs include processes, impacts, and complete feedback cycles in the climate system. Simulations of past climates with comprehensive ESMs provide information about the dynamical mechanisms that could lead to hydrological and thermal periods that deviate from average climate conditions. These climate conditions may be caused by the influence of external factors, such as changes in solar and volcanic activities, or by purely internal variations in oceanic and atmospheric circulation.

Simulations carried out with ESMs generate possible climate evolutions compatible with the prescribed configurations of the external drivers (solar variations, volcanic eruptions). However, this simulated evolution also depends on initial conditions, which cannot be known with perfect precision: even the best instrumental measurements cannot generate the exact initial conditions required for a model to undergo changes with the same timing as the real climate system. Therefore, the reconstructed and simulated evolutions do not need to agree in the timing of climate events. We selected the CCSM4 and MPI-ESM-P model outputs from the CMIP5 simulations (Taylor *et al*. 2012) that start in 850 CE for comparisons with reconstructions and natural proxy-based climatic evidence for the Crusader, the Mamluk, and the Celâlî period. Both simulations have the highest spatial horizontal resolution among the CMIP5 simulations. The fine spatial resolution is important, especially for hydrological investigations, so that the underlying physical processes can be simulated more realistically.

The CCSM4 model consists of an atmospheric component CAM4 with 26 vertical levels and a horizontal resolution of 0.9°-1.25° that is coupled with the ocean model POP. The ocean model has a variable horizontal resolution of 1.1°, which increases from 0.54° at 33° N/S to 0.27° at the equator, and 60 vertical levels. The land model used is the CLM4^1^ for which only one simulation is available. The advantage of the simulation is its extraordinarily high spatial resolution for a global ESM, which corresponds to 80 km longitude × 140 km latitude over the Mediterranean region.

The MPI-ESM-P model consists of the atmospheric model ECHAM6 with horizontal resolution 1.85° × 1.85° that is approximately 160 km longitude × 200 km latitude over the Mediterranean region and 47 vertical levels. The model is coupled with the ocean model MPI-OM (of a bi-polar curvilinear grid: 1.5° horizontal resolution with 40 vertical levels). The simulations incorporated changes in external forcing parameters (i.e., changes in Earth’s orbital configuration, volcanic eruptions, solar variations, and anthropogenic changes in the composition of the atmosphere and land use change; IPCC 2013) following the Paleoclimate Modelling Intercomparison Project Phase III (PMIP3) protocol (Schmidt *et al*. 2012).

### Comparing Climate Model Simulations with Proxy Based Reconstructions

Palaeoclimate information is important for evaluating the models’ ability, as it provides the only source of validation. Temperature and hydro-climatic changes are used to investigate responses to external climatic forcings (e.g., solar, volcanic, greenhouse gases, land-use/land-cover changes) and to better quantify the internal variability of the climate system (Smerdon *et al*. 2017). Climate models provide complete information on many climate variables and processes (for instance, precipitation, atmospheric circulation, and temperature) that are internally consistent with the model physics. However, a quantitative comparison between the output of climate models and proxy-based reconstructions is not a straightforward step due to the uncertainty in both the models (e.g., initial conditions, external forcings reconstructions) and the proxy-based reconstructions (e.g., proxy records, observations, applied methodologies – see above). Moreover, even a perfect model that is forced with the real external forcings of the past will ultimately show a different climate trajectory when compared to reality due to the slightest difference of the initial state of the models (e.g., changes related to the state of the ocean temperature and the sea ice concentration over high latitudes).

Major sources of uncertainty in climate models include the limitations to spatial resolution that cause errors in the simulation of topographical effects and finer-scale processes, the parameterization^2^ of small-scale physical processes, the tuning to modern-day climate, and the selection of (uncertain) forcing time series. Hydrological variables are controlled by processes that usually occur at scales smaller than the grid boxes of the climate models, and therefore these must be parameterized. Precipitation amount is a complex target for proxy-model comparisons due to highly localized influences such as topography and small-scale dynamics that are not sufficiently well resolved (Stephens *et al*. 2010; Davini and Cagnazzo 2014).

Proxy-model comparisons are effective when they leverage the complementary strengths of both information sources and are viewed as a comparison between two independent sources of information used to test process-based hypotheses (Smerdon *et al*. 2017). Therefore, comparisons should be considered within a framework in which proxy-derived reconstructions and simulations compensate each other’s deficiencies. Smerdon *et al*. (2017) provide recommendations for integrating proxies and model simulations. Given the natural variability in the climate system and in the model simulations, it is unrealistic to expect congruent chronologies in proxy-based reconstructions and model simulations of past climates. Direct time comparisons can be misleading, especially if events in the palaeoclimate record are affected by a high degree of internal variability rather than exogenous forcing (Gómez-Navarro *et al*. 2015; Smerdon *et al*. 2017). Internal variations will evolve differently from one model simulation to the next unless initial conditions are identical, and amplitudes of decadal and longer timescale modes of climate variability differ among model frameworks (Smerdon *et al*. 2017). Despite their usually low temporal correlation, proxy-model comparisons can still be meaningful when they focus on underlying physical processes and mechanisms.

As outlined above, different levels of complexity and approaches can be used in the context of model-proxy comparison. In order to provide a general picture, we perform a classical model-data comparison using the direct output fields of the model simulations in conjunction with the climate reconstructions. We compare hydro-climate proxies (Fig. 2), reconstructed temperature, precipitation, and drought fields with simulations from the CCSM4 and MPI-ESM-P models for the periods 1220–1250, 1260–1450, and 1580–1610 CE. We examine statistics of means, variability and trends, and analyze important processes, including simulated responses to changes in internal and external forcing. Furthermore, to avoid the potentially large uncertainties associated with the coarse spatial resolution of the climate models, our reconstruction-model comparisons focus on dynamics and hydro-climate features with large spatial scales that span the average of multiple grid boxes.

### Simulated Extended Winter Temperature and Precipitation since 850 CE

**Fig. 3:**
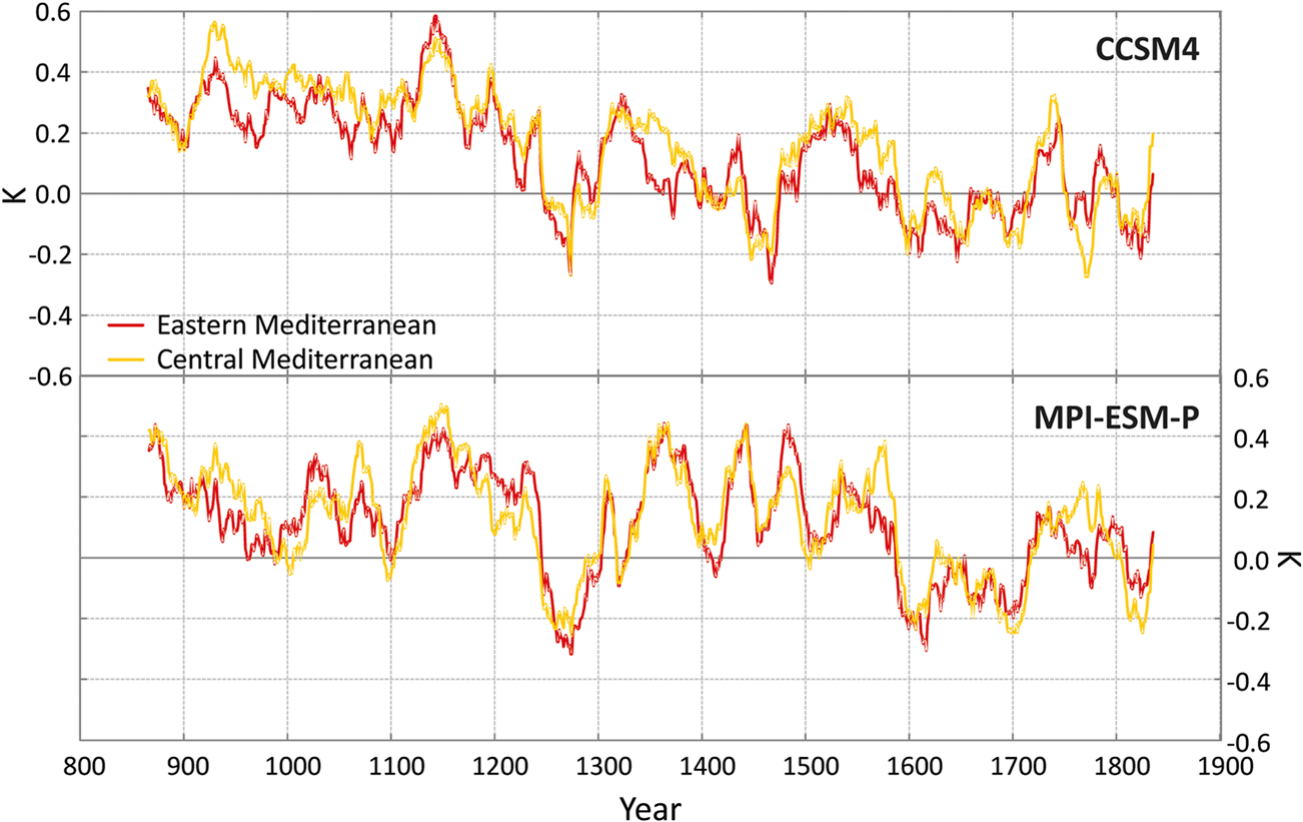
Simulated extended winter temperature anomalies (K) for the Eastern (20–40° E, 30–45° N, red lines) and Central (10–20° E, 30–45° N, yellow lines) Mediterranean for the CCSM4 and the MPI-ESM-P models from 850 to 1850 CE. The curves are filtered with a 30-year running mean

**Fig. 4:**
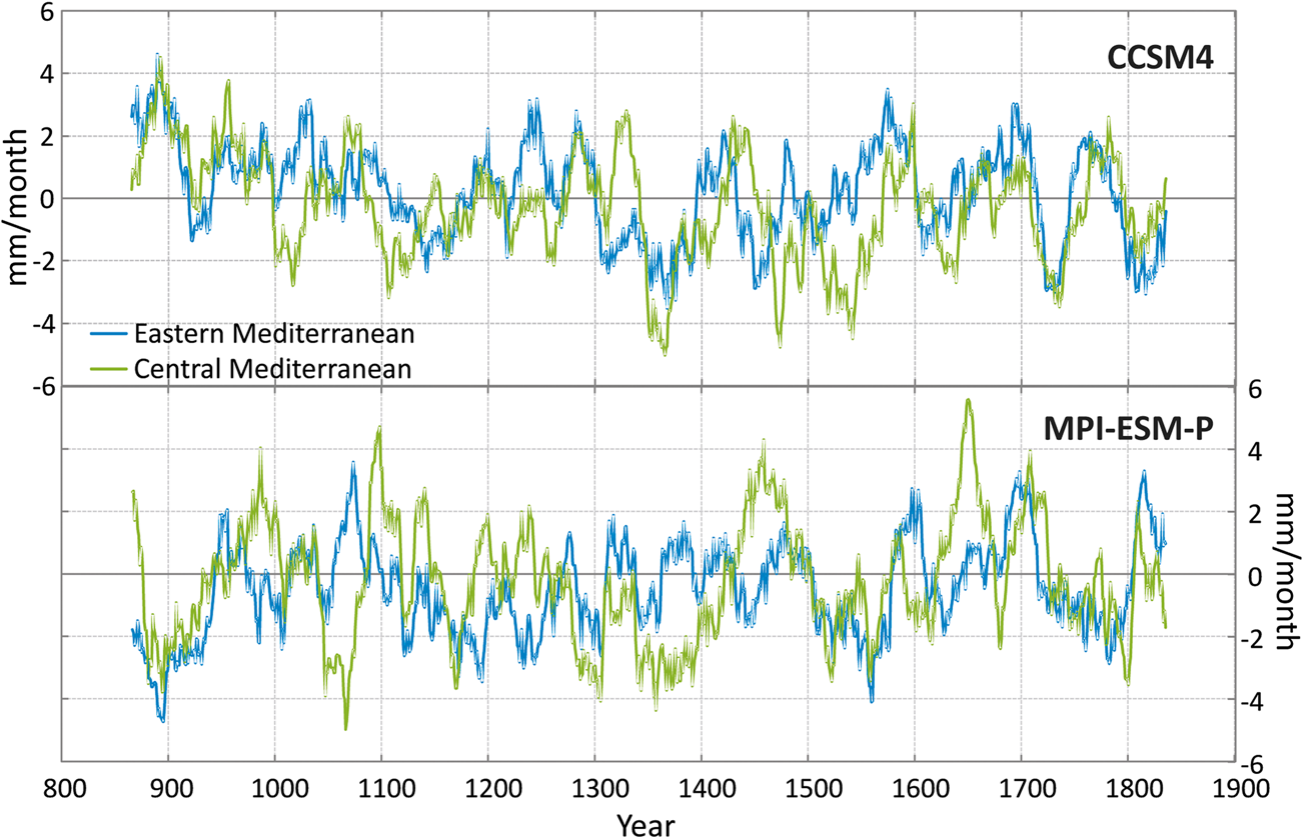
Simulated extended winter precipitation anomalies (mm/month) for the Eastern (20–40° E, 30–45° N, blue lines) and Central (10–20° E, 30–45° N, green lines) Mediterranean for the CCSM4 and the MPI-ESM-P models from 850 to 1850 CE. The curves are filtered with a 30-year running mean

We averaged the model results for near-surface temperature and precipitation for the extended winter period, October to March (Xoplaki *et al*. 2016), over the Central (10°–20° E, 30°–45° N) and Eastern (20°–40° E, 30°–45° N) Mediterranean for the period 850–1850 CE (Figs. 3 and 4). The spatially averaged variables are presented as deviations (anomalies) from the mean conditions of the period 1651–1850 CE, which we selected as the common reference period for the reconstructed fields and model simulations. To better visualize multi-decadal variations, the original curves are filtered with a 30-year running mean.

The extended winter temperature anomalies for the Eastern and Central Mediterranean are in very good agreement between the two models in terms of their temporal evolution. Both models generally show warmer conditions (with respect to the 1651–1850 CE climate) until the mid-thirteenth century. According to Gogou *et al*. (2016), who report an increase in sea surface temperatures from ca. 850 to 950 CE and from ca. 1100 to 1300 CE, this is in agreement with evidence from sea surface temperature variations and palaeooceanographic/palaeoenvironmental changes over the North Aegean Sea. The period between the Oort (1040–1080 CE) and Wolf (1280–1350 CE) solar minima coincides with the MCA, which is generally characterized by warmer climate conditions in the area (Diaz *et al*. 2011). The warmer conditions during parts of the MCA might be related to a reduction in volcanic activity and/or higher solar activity. Both model simulations show a significant reduction of cold season temperatures around the middle of the thirteenth century related to the great Samalas volcanic eruption and other tropical volcanic eruptions of that period (Sigl *et al*. 2015; Guillet *et al*. 2017). Both models show warmer conditions again at the beginning of the fourteenth century. In the CCSM4 model, a cooling trend is visible, with minimum values at the end of the fifteenth century.

The picture of the extended winter precipitation is more complex (Fig. 4) due to the underlying smaller-scale physical phenomena that control precipitation variability. The two simulations present different precipitation realisations for the last millennium. Highly variable periods of synchronous and asynchronous humid and dry periods between the two sub-basins can be found in both simulations. The two sub-basins within MPI-ESM-P model are generally in anti-phase until the end of the seventeenth century.

The varying temporal evolution between the two models is largely a manifestation of the strong internal regional variability which is unaffected by changes in solar, orbital, and volcanic activity. This is visible for instance during the Crusader period (1095–1290 CE), when the MPI-ESM-P model shows a general drying trend in the Central and Eastern Mediterranean, whereas the CCSM4 model does not reveal a long term change for these regions.

A general conclusion from the model results relates to the better agreement in the temporal evolution of the simulated temperatures in the Mediterranean compared to precipitation. The important pacemakers are volcanic eruptions, especially the larger ones that leave their imprint in the temperature time series of both model simulations. For winter precipitation the two model simulations show no coherent evolution, a consequence of the higher complexity of processes and dynamics that control precipitation and the large spatial heterogeneity of precipitation changes.

## Climate Conditions in the Study Periods

### Crusader Period, 1095–1290 CE

**Fig. 5:**
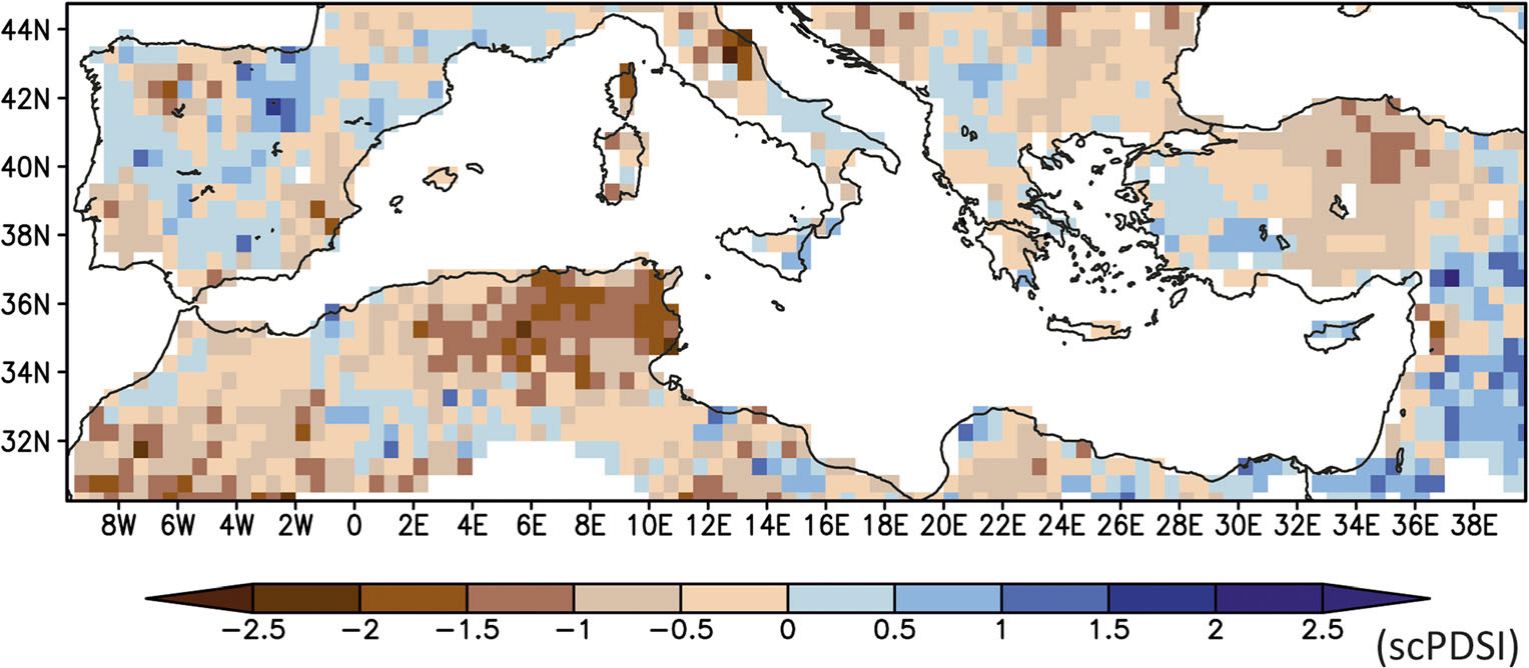
Mean scPDSI for the period 1220–1250 CE (data from OWDA, Cook *et al*. 2015)

The strategic and political situation of the Crusader States in the Levant was precarious from their establishment in the late eleventh century, and they disintegrated within two centuries. Although climate in the Levant was wetter and more amenable to local agriculture during the early Crusader period, with the exception of sugarcane and grapevines, in which they invested heavily (Prawer 1973), the elites preferred to import large quantities of grain and other staples rather than increase local production through existing structures. The Crusader kingdoms relied more heavily on such imports during times of increased external political pressure, but after the middle of the twelfth century they imported food even in the best of times (Richard 1985). Especially in the later thirteenth century when the extent of their lands shrank, they relied increasingly heavily on imported provisions from the Central Mediterranean and Sicily in particular (Pryor 1988). This was the case also during the period 1220–1250 CE, when the drought reconstructions by Cook *et al*. (2015) show generally dry conditions over the Central Mediterranean (Fig. 5) in agreement with the reconstruction of the Pergusa Lake (Fig. 2). Over Anatolia, the same reconstruction reveals dry conditions, which is not in agreement with the Nar Lake record that indicates generally wetter conditions (Fig. 2). For the Levant region/Jordan Valley area, the drought reconstruction shows wetter conditions over the mainland that are only supported by the effective moisture reconstructions from the Jeita Cave but not from the Kocain Cave (Fig. 2). As mentioned above, the stalagmite δ^18^O records can have chronological uncertainties ranging from a few years to several decades; thus, a direct comparison is difficult at those short time scales.

**Fig. 6:**
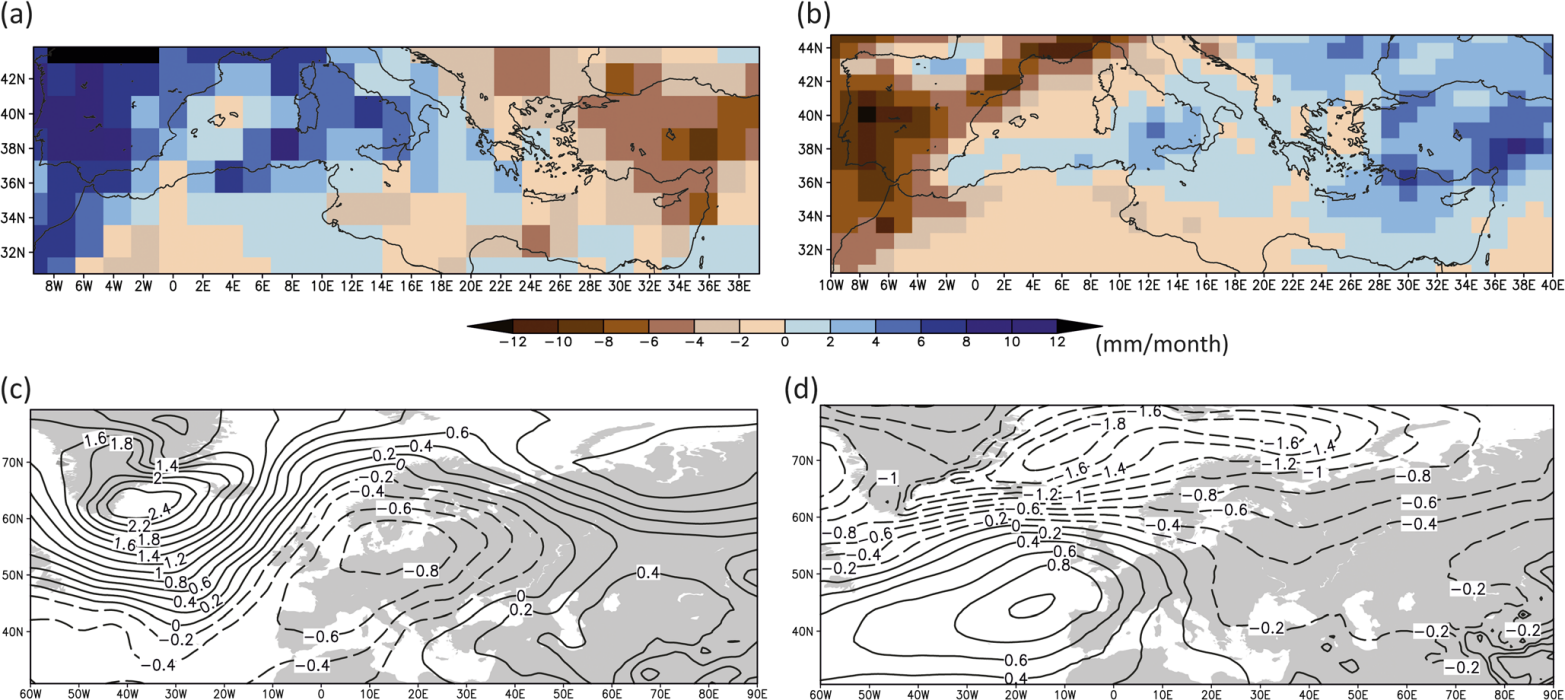
Simulated extended winter precipitation anomalies in the Mediterranean during the period 1220–1250 CE (upper panel, in mm/month) and associated sea level pressure conditions over the Euro-Atlantic area (lower panel, in hPa): (**a**, **c**) MPI-ESM-P, (**b**, **d**) CCSM4. The anomalies are calculated with respect to the reference period 1651–1850 CE

The CCSM4 and MPI-ESM-P simulations show different trajectories for the mean hydrological conditions over the Mediterranean (Fig. 6a, b). The MPI-ESM-P model reveals a dipole pattern with wetter conditions over the Western and Central Mediterranean and dryness over the eastern basin, and thus provides a potential climate trajectory that supports the role of the Kingdom of Sicily as an important supplier of provisions for the Crusader Levant. This precipitation distribution is linked to anomalous low-pressure conditions^3^ stretching from the subtropical Atlantic over large parts of the Mediterranean and Europe towards Western Russia. The Eastern Mediterranean is located in a transition zone of the dipole towards higher sea level pressure conditions (compared to the reference period 1651–1850 CE, Fig. 6c) that is connected with drier conditions.

The CCSM4 model also shows a west-east dipole precipitation pattern, with its sign reversed compared to the MPI-ESM-P similar to the proxy records and the drought reconstruction (Figs. 5 and 6a, b). The dryer conditions over the Western Mediterranean and the wetter climate over the Eastern basin are associated with strong anomalous subtropical high-pressure conditions^4^ extending towards Central Mediterranean and Western Europe (Fig. 6d).

The model results (Fig. 6) suggest that the precipitation and atmospheric circulation anomalies during this period were not caused by external forcings (i.e., volcanic, orbital, solar), since the two simulated hydro-climate patterns clearly disagree. The simulated hydro-climatic anomalies may therefore be caused by the models’ internal variability,^5^ which is especially pronounced at regional spatial scales and sub-decadal time scales (Hawkins and Sutton 2009; Deser *et al*. 2014; Gómez-Navarro *et al*. 2015), and they could have occurred at any period within the last millennium. Climate models simulate the physical mechanisms and processes of the climate system and within its components and the coupled interactions among them. In the absence of external forcing, the temporal evolution of the climate system is influenced only by its own internal dynamics and thus for each climate model can follow a different trajectory.

Another issue that further complicates the interpretation of results based on the simulations with respect to hydro-climate is whether the models can mimic the complex small- to meso-scale^6^ atmospheric circulation patterns that influence the Mediterranean (e.g., Lionello *et al*. 2016). A realistic simulation of these systems (e.g., the Genoa and Cyprus cyclones) is crucial for a proper estimation and assessment of any past and future change in the hydrological variability of the Mediterranean in general. Furthermore, these systems are generated or regenerated over the Mediterranean region and the frequency of those systems over the course of a year may control a large portion of the total precipitation amounts and the hydrological variability, especially over the central and eastern parts of the basin, where the highest frequency of cyclone tracks is observed (Lionello *et al*. 2016).

The palaeoclimate proxy records (Figs. 2 and 5) and, to a degree, the model simulations (CCSM4, Figs. 4 and 6b) show that during our study period of 1220–1250 CE, the Southern Levant was experiencing relatively wet to normal conditions (Fig. 2, Nar Lake, caves Soreq, Jeita and Gejkar, OWDA Eastern Mediterranean), while Italy and Sicily – major grain exporters – were primarily experiencing a warmer and dry to normal climate (Fig. 2, OWDA Central Mediterranean, Pergusa Lake). Such conditions would make it more difficult to develop the intensive cereal cultivation in the Central Mediterranean that would be adequate for export over and above local requirements. In other words, southern Italy and Sicily supplied grain to the Crusader States in the Levant despite the fact that the hydro-climatic situation was relatively good for cereal cultivation in the Southern Levant, but unfavourable in the Central Mediterranean. This demonstrates the extent to which the patterns of resource management, trade, and agriculture in complex pre-industrial societies were independent of climatic trends, and instead shaped by political, economic, religious, and cultural factors.

### Mamluk Period, 1260–1516 CE

The Mamluk state was established during the thirteenth century in Egypt and the Levant by imported slave-soldiers, who maintained and expanded existing agricultural patterns of intensive land use and irrigation. The Jordan Valley is a region of great importance with respect to water resources and agricultural potential in the area (Wilkinson 2003). The Mamluks initially exploited the generally beneficial precipitation conditions of their period to produce sugar as a cash crop, exporting it throughout the Mediterranean (Orland 2009; Neumann *et al*. 2010; Litt *et al*. 2012; Preiser-Kapeller 2015; Flohr *et al*. 2017) in spite of recurring droughts and other natural and human induced calamities (Raphael 2013).

The decades after the first wave of the Black Death in 1346 CE were characterised by a destabilisation of Mamluk rule, civil wars, and the loss of imperial control over territories (Van Steenbergen 2006), leading to a decrease in the productivity of the sugar industry (Haarmann 1994) and loss of overseas markets (Haarmann 1994; Ouerfelli 2008). Proxy data indicate a general and large scale trend towards drier conditions (see Fig. 2, Jeita and Soreq caves along the Jordan Valley, Nar Lake to the north, and Gejkar Cave farther northeast) and cooler winters across the region (Kocain Cave denoting an enhanced snow cover, Fig. 2), which in combination with recurring waves of plague, may have impeded a recovery of agricultural productivity in the region (Kareem 2000; Neumann *et al*. 2010; Kaniewski *et al*. 2011; Litt *et al*. 2012; Flohr *et al*. 2017).

**Fig. 7:**
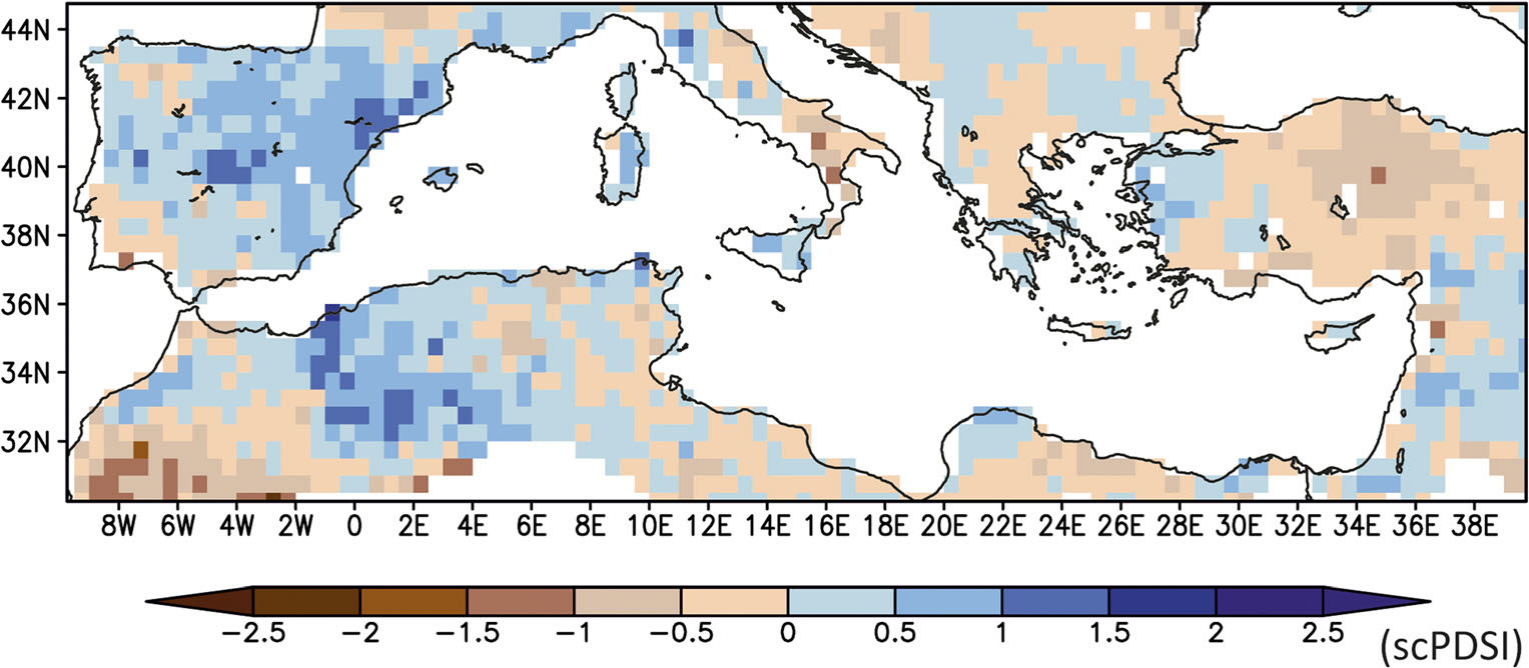
Mean scPDSI for the period 1260–1450 CE (data from OWDA, Cook *et al*. 2015)

Drought field reconstructions (Cook *et al*. 2015), in agreement with the proxy records, show relatively humid conditions over the Jordan Valley (Fig. 7) that facilitate water-intensive sugarcane cultivation through the extensive irrigation systems developed in the early Mamluk period. Irrigated agriculture in Southern Levant is influenced by the climatic conditions due to the high interannual precipitation variability and recurring droughts as well as the Jordan River’s extremely variable annual discharge rate (Chenoweth *et al*. 2011). Following the relatively humid conditions of the early Mamluk period, independent proxy information indicates a gradual change towards drier conditions starting in the early fourteenth century and speeding up from the mid-fourteenth century onwards (Kaniewski *et al*. 2011), and this finding is partially supported by evidence from speleothems in the greater study area (Caves Jeita and Gejkar, Fig. 2). A distinct and abrupt change from more humid to more arid conditions occurred at the end of the fourteenth century to the north, in the Nar Lake record (Fig. 2).

**Fig. 8:**
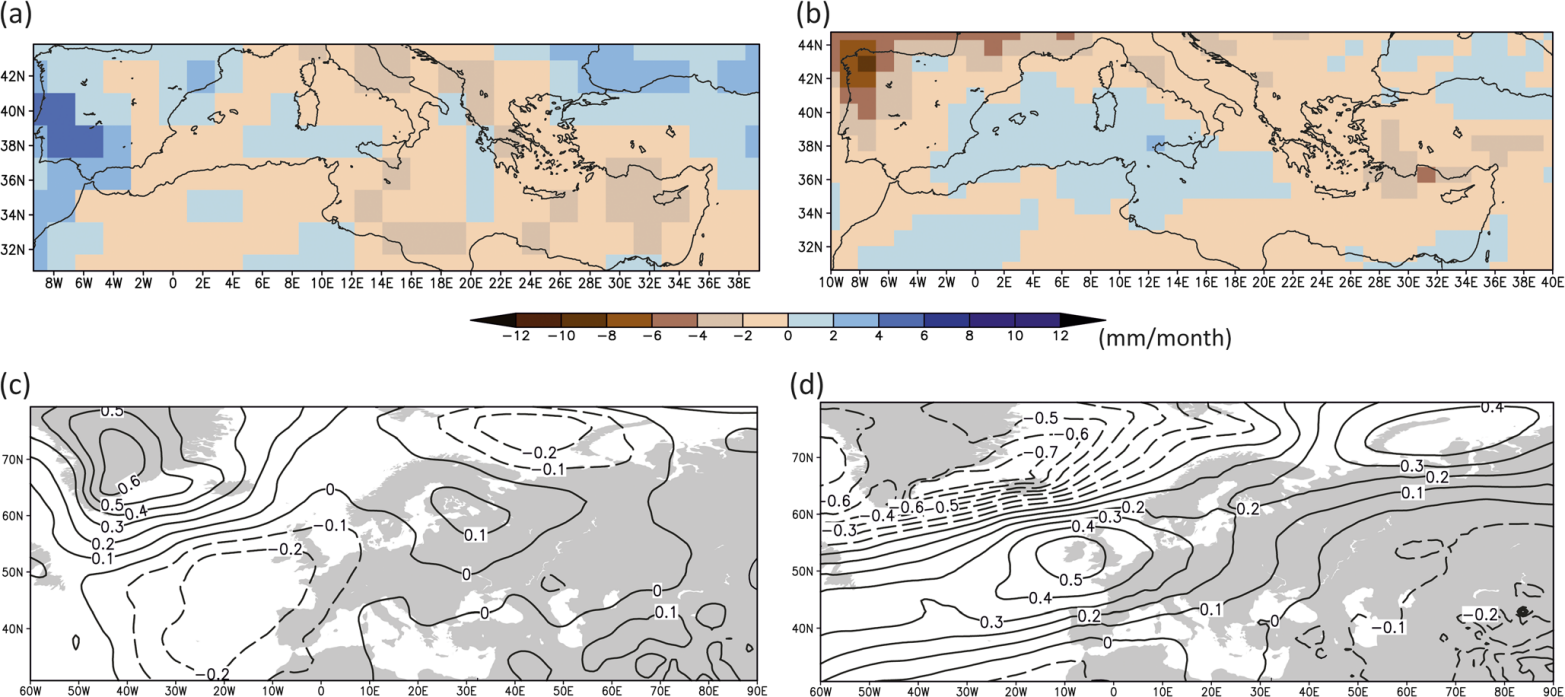
Simulated extended winter precipitation anomalies in the Mediterranean during the period 1260–1450 CE (upper panel, in mm/month) and associated sea level pressure conditions (anomalies) over the Euro-Atlantic area (lower panel, in hPa): (**a**, **c**) MPI-ESM-P, (**b**, **d**) CCSM4. The anomalies are calculated with respect to the reference period 1651–1850 CE

Similar to the period ca. 1220–1250 CE, the different climate trajectories in the simulated hydro-climate patterns of the two models suggest that the precipitation anomalies for the period 1260–1450 CE (Fig. 8a, b) are not linked to the impact of external forcing but are governed by the internal variability of the climate system and smaller scale atmospheric circulation patterns that influence the Mediterranean. An interesting feature is the dipole precipitation pattern over the Eastern Mediterranean, with slightly wetter conditions to the north and drier conditions over the southern areas. However, the sea level pressure patterns (Fig. 8c, d) reveal no clear physical pattern over the Eastern Mediterranean that can explain this dipole pattern in a consistent manner. For the Northwest Mediterranean, the wetter and drier conditions can be explained by a decrease and increase in sea level pressure for the CCSM4 and MPI-ESM-P model simulations, respectively. Still, the response is opposite for both model simulations, which underlines the complexity of the changes in precipitation, even over longer time scales.

The comparison between the model simulations (Fig. 4, CCSM4), proxy data (Fig. 2, Jeita Cave, Nar Lake, Kocain Cave, Gejkar Cave), historical sources, and archaeological evidence indicates that, in general, climatic conditions supported the continuation or even expansion of water-intensive sugar production in the Jordan Valley during the early Mamluk period (from 1260 CE up to the early fourteenth century). Later, changes from more humid to more arid conditions indicated in the model simulation (Fig. 4, CCSM4) and in proxy data, though not fully synchronous, (Fig. 2, Jeita Cave, Nar Lake, Kocain Cave, Gejkar Cave) overlapped with the outbreak of plague epidemics and accompanying socio-political unrest from the mid-fourteenth century onwards (Campbell 2016), increasing the vulnerability of this resource-intensive agricultural model. Nevertheless, even in the face of changing conditions, Mamluk elites attempted to persist in established agricultural policies and investment strategies in order to maintain a stable level of revenue extraction. Although this approach resulted in an enduring demographic and economic depression throughout the fifteenth century, it also illustrates the endurance of a cultural logic rooted in established specific belief-systems.

### The Celâlî Rebellion, 1580–1610 CE

**Fig. 9:**
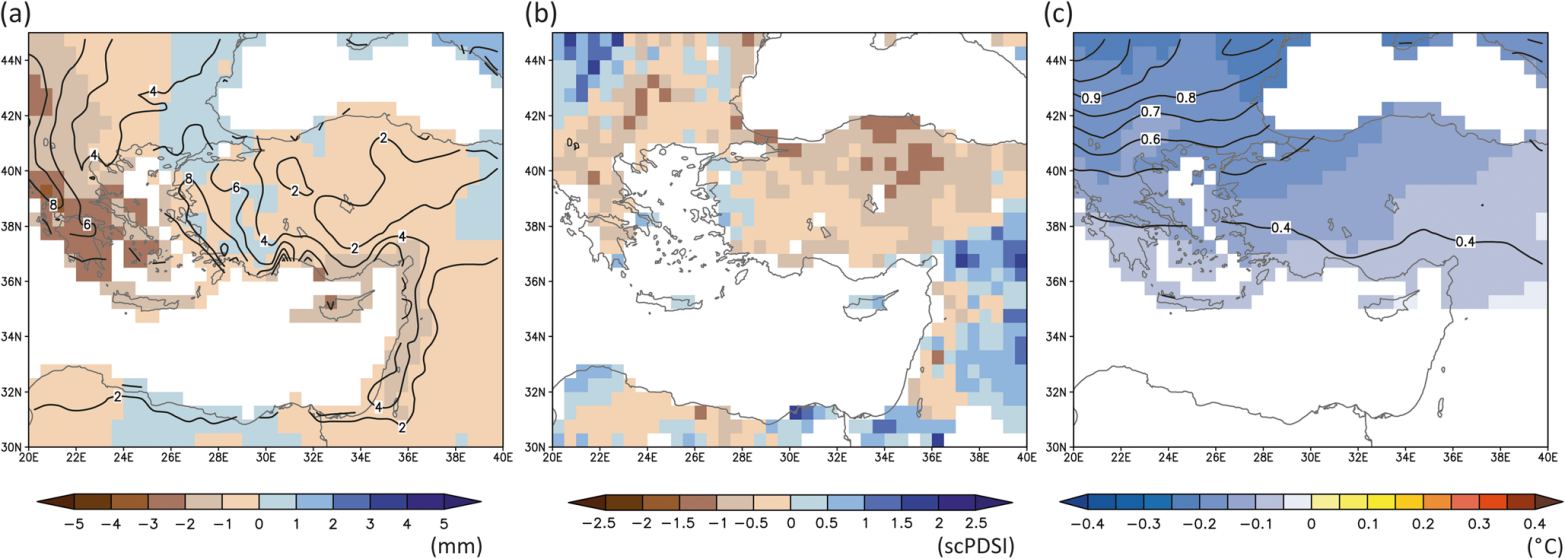
The period 1580–1610 CE – the Celâlî Rebellion: (**a**) wet season precipitation deviations (with respect to the period 1651–1850 CE) with associated standard deviation (solid lines; in mm) (data from Pauling *et al*. 2006); (**b**) mean scPDSI (data from OWDA, Cook *et al*. 2015); (**c**) wet season temperature deviations (with respect to the period 1651–1850 CE) with associated standard deviation (solid lines; in °C) (data from Luterbacher *et al*. 2004; Xoplaki *et al*. 2005)

**Fig. 10:**
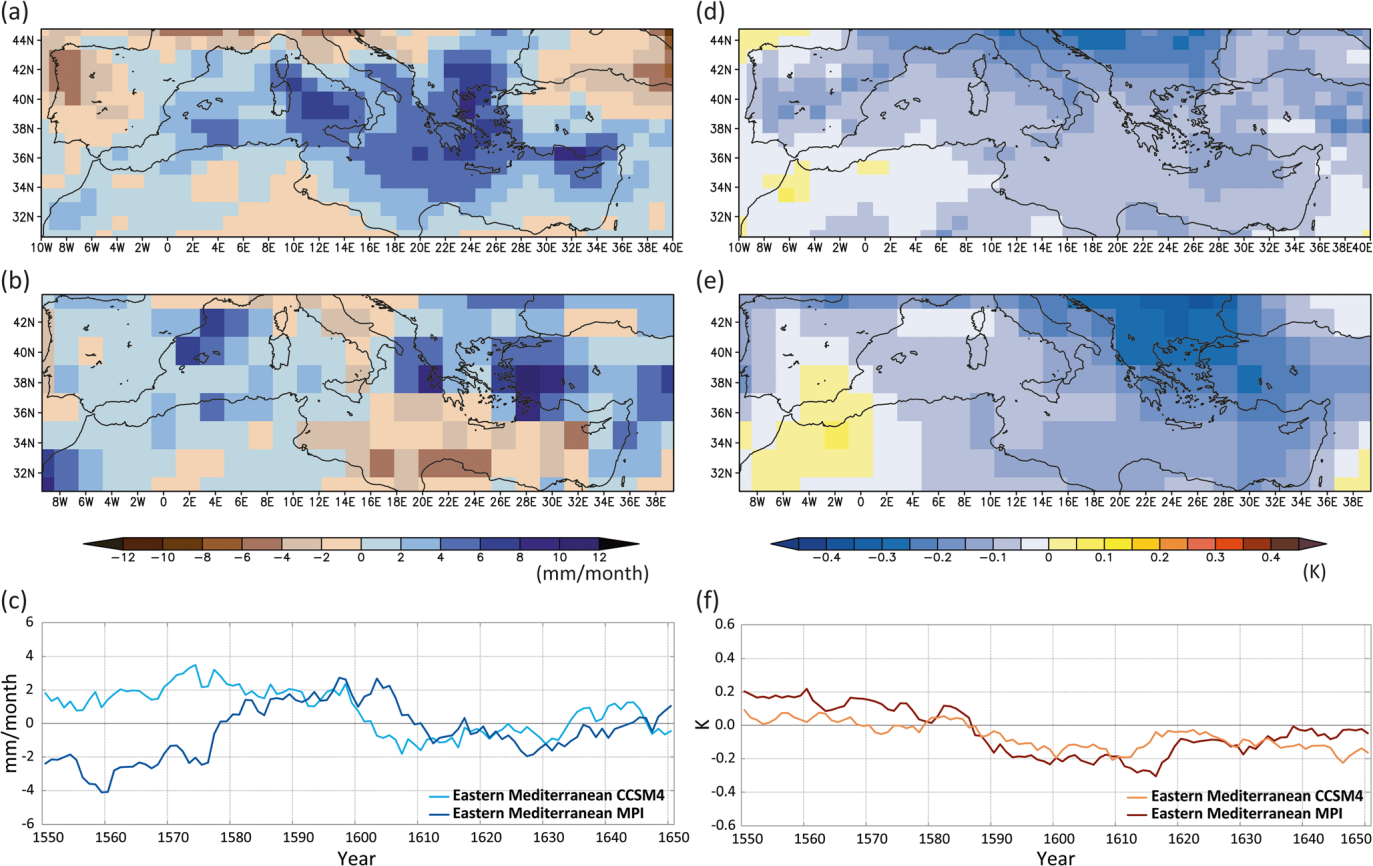
Simulated extended winter precipitation (left panel, in mm/month) and temperature (right panel, in K) anomalies in the Mediterranean during the period 1580–1610 CE: (**a**, **d**) CCSM4, (**b**, **e**) MPI-ESM-P, (**c**, **f**) Temporal evolution of simulated precipitation and temperature anomalies over the Eastern Mediterranean (20–40° E, 30–45° N). The anomalies are calculated with respect to the reference period 1651–1850 CE

Between 1580 and 1610 CE the Ottoman Empire underwent a political and economic crisis (“the Ottoman Little Ice Age crisis”) triggered by multiple environmental and human stresses (see below and Figs. 9 and 10). Historical sources refer to periodic cold and dry weather conditions during the 1580s, followed by a major drought in 1591–1596 CE in Anatolia and the Southern Balkans (White 2011; Izdebski A, Mordechai L, and White S. The costs of resilience in premodern societies. Human Ecology, under review) that led to harvest failures and then famine in the affected areas. Combined with cold winters, it likely contributed to the outbreak of a major epizootic, probably rinderpest. In this context, imperial demands for sheep from depleted rural flocks triggered a provincial revolt in Anatolia known as the Celâlî Rebellion (1596–1610 CE) (White 2011, 2017). Unusually cold winters and variable precipitation continued into the first decade of the seventeenth century. Positive feedback between famine, violence, population displacement, and infectious disease led to population losses of 50% or more in parts of the empire between the 1580s and 1630s (Özel 2016).

The multi-proxy precipitation and temperature reconstructions by Pauling *et al*. (2006) and by Luterbacher *et al*. (2004) and Xoplaki *et al*. (2005) for the 1580–1610 CE wet season (autumn-winter) deviations from the reference period 1651–1850 CE agree with the historical sources, and indicate an increase in the frequency of severe droughts (Fig. 9a) and colder conditions (Fig. 9c), respectively. The precipitation reconstructions reveal a general reduction in autumn/winter precipitation over the Eastern Mediterranean compared to the reference period (1651–1850 CE; Fig. 9a). The driest areas are located over Greece where the standard deviation is also largest. A similar spatial pattern shows the summer scPDSI reconstruction by Cook *et al*. (2015) (Fig. 9b). The two hydro-climate patterns differ over the Northern Balkans and the Levant. The temperature reconstructions by Luterbacher *et al*. (2004) and Xoplaki *et al*. (2005), which do not include any proxy information from the area, show a distinctively cold 30-year period from 1580 to 1610 CE compared to the reference period (Fig. 9c). The largest temperature deviations appear throughout the Balkans, the area with strongest temperature variability. Independent winter-to-spring temperature reconstructed from tree rings support the cooler conditions in parts of Turkey (Heinrich *et al*. 2013, not shown). The high temporal resolution of the Nar Lake record (Fig. 2) enables identification of decadal to multi-decadal droughts, with increased aridity (more positive δ^18^O calcite values) at the end of the sixteenth century, coincident with the Celâlî Rebellion.

The 1580–1610 CE wet season CCSM4 and MPI-ESM-P simulated precipitation and temperature deviations from the reference period 1651–1850 CE (Fig. 10) generally show a wetter 30-year period over the Central and Eastern Mediterranean, in contrast to climate reconstructions and written historical evidence (Fig. 9). The temporal evolution of the Eastern Mediterranean precipitation (Fig. 10c) indicates a distinct drying trend at the end of the sixteenth century, slightly later than the historical evidence and reconstructions show. The models agree on the generally cooler period 1580–1610 CE over the Eastern Mediterranean (Fig. 10d, e) with similar temperature departures. The two models agree on the cooling trend that starts in the 1590s and lasts until the mid-1610s (Fig. 10f).

**Fig. 11:**
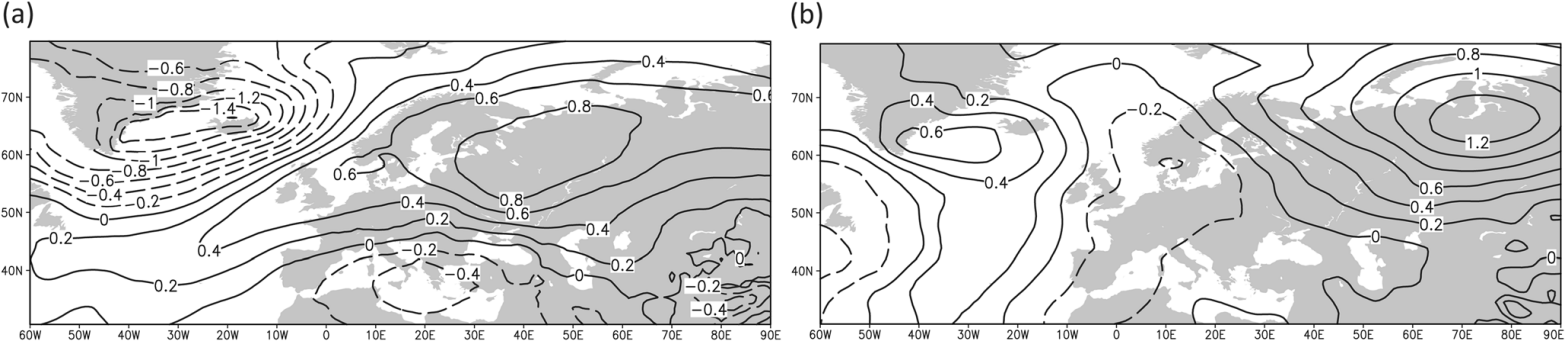
Simulated extended winter sea level pressure anomalies (with respect to the period 1651–1850 CE) for the period 1580–1610 CE (in hPa/month) from (**a**) the CCSM4 and (**b**) the MPI-ESM-P models

The wetter and colder conditions of the CCSM4 model for the period 1580–1610 CE are related to a positive sea level pressure anomaly located over Western Russia and a lower pressure anomaly over the Central Mediterranean (Fig. 11a). This pressure distribution caused a persistent cold easterly airflow at the southern margins of the continental high, supported by latent and sensible heat^7^ flux due to the warmer Mediterranean Sea connected with cyclogenesis and higher precipitation values.

The model simulations, therefore, suggest that the temperature evolution is more closely related to the impact of external forcings, possibly the strong volcanic eruptions that occurred in this period (see Sigl *et al*. 2015) and the negative trend in the solar activity (Steinhilber *et al*. 2009). The cluster of strong volcanic eruptions might have been an initial trigger for the anomalous temperature conditions amplified by the weak subpolar gyre in the North Atlantic and the associated changes in atmospheric circulation over Europe and the Mediterranean area (Moreno-Chamarro *et al*. 2017). This impact is not found in the simulation of precipitation, as this is more related to anomalies of atmospheric circulation, which is to a large degree independent of changes in external forcings, at least in the context of the last millennium (Gómez-Navarro and Zorita 2013). Atmospheric circulation is generally less strongly tied to the direct radiative impacts of external forcing than surface temperatures are. Climatic conditions over the North Atlantic after major volcanic eruptions are characterized by a post-volcanic Northern European winter warming (Robock and Mao 1992; Kirchner *et al*. 1999; Fischer *et al*. 2007; Zanchettin *et al*. 2013). This phenomenon is related to dynamical processes that link stratospheric and tropospheric vortices and increase the probability of a positive state of the North Atlantic Oscillation that is an important driver of the European winter climate variability. During the positive state of the North Atlantic Oscillation, Northern Europe in particular receives more humid air masses from the Atlantic Ocean. For the Mediterranean region, a positive phase of the North Atlantic Oscillation is related to a more complex hydrological pattern depending on location (e.g., Dünkeloh and Jacobeit 2004; Xoplaki *et al*. 2004; Roberts *et al*. 2012). The impact on winter temperature over the Mediterranean area is thus less discernible (e.g., Wanner *et al*. 2001).

Insofar as this palaeoclimate modelling reveals spatial and seasonal patterns of drought and temperature variability not captured in the written and proxy evidence, it also underlines the significance of local human factors and historical contingencies in mitigating climate’s impacts on past societies. The Balkans – and particularly Greece – experienced climate anomalies during ca. 1580–1610 CE that were at least as extreme as those in central Anatolia. Written and archaeological records confirm that the former area did suffer from episodes of famine and violence, leading to population loss by the early seventeenth century. In central Anatolia, similar environmental stresses are found to have also triggered rebellion, leading to mass migration and political and economic destabilization across the Ottoman Empire (White 2011). Several factors may have been responsible for Central Anatolia’s vulnerability at the outbreak of crisis, including the area’s population growth during the preceding decades, its agricultural systems heavily focused on winter wheat and barley, and its role in providing additional taxes and provisions for the empire during military campaigns (White 2011).

## Conclusions

We analysed and interpreted the hydrological and environmental proxy evidence, spatial temperature, precipitation and drought reconstructions in tandem with state-of-the-art model simulations for three periods: the Crusader Levant (1095–1290 CE), the Mamluk regime in Transjordan (1260–1516 CE), and the Ottoman Little Ice Age crisis (1580–1610 CE). In all three periods, environmental and climatic stress tested the resilience of past complex societies in the Eastern Mediterranean.

The following conclusions can be drawn from this study:

- Variations in precipitation and drought over the Eastern Mediterranean at the multidecadal timescale are driven by internal climate dynamics and cannot be explained in terms of external forcings for the studied periods. They also show a larger spread of the models’ trajectories and patterns. Other model simulations may display similar hydro-climatic conditions, trends, and variations but again with different timings. Therefore, no agreement can be expected with the timing of hydro-climate events between the model simulations, the proxy records, and the proxy-based reconstructions.
- Despite the lack of agreement between model simulations and proxy-based reconstructions in the temporal occurrence of hydro-climatic fluctuations over the first 500 years of the past millennium, ESMs provide important information on the dynamic mechanisms and underlying processes that could lead to anomalous hydrological and thermal periods in the past. For the Mediterranean region in particular, great challenges remain with respect to the proper simulation of small to mesoscale circulation patterns that influence hydrological variability, including drought and wet spells.
- Overall, archive-specific limitations of proxy data sources (seasonal bias, temporal resolution and dating uncertainties) combined with different regional climatic characteristics make it currently impossible to trace basin-wide hydro-climatic changes with confidence, which also limits our ability to establish firm causal links between hydrological changes and societal transformations.
- Integrated analysis of palaeoclimate proxies and model simulations sheds additional light on our understanding of past climate change and its societal impacts. It also reveals the limited extent in which climate trends themselves determine actions and patterns of behaviour – economic or political – in complex historical societies. Our research also demonstrates that climate-related crises are not the work of nature alone: they occur in the context of historically specific vulnerability to climate change developed by certain societies at certain moments in time, as demonstrated for all three case studies. Consequently, our research emphasises the need to further study the societal dimension of climate change in the past, which is essential given today’s political realities, when the impact of climate change on human societies remains of topic of debate and its effects tend to be ignored.

## Footnotes

1 A comprehensive description of the simulation of the last millennium can be found in Landrum *et al*. (2013).

2 Parameterization is a technical process that provides an approximation of the real process in the climate model to account for small-scale processes below the grid point scale.

3 Anomalous low sea level pressure conditions denote a higher frequency of low-pressure systems passing through the study area compared to the reference period.

4 Anomalous high-pressure conditions denote a persistence of high pressure/anticyclonic conditions over the area compared to the mean conditions during the reference period.

5 Internal climate variability refers to variations in the mean and other statistics of the climate that are due to the internal climate system processes and thus to the internal variability of the major climate system components (atmosphere, hydrosphere, cryosphere, land surface, and biosphere) and their coupled interactions.

6 “Meso-scale: Pertaining to atmospheric phenomena having horizontal scales ranging from a few to several hundred kilometres, including thunderstorms, squall lines, fronts, precipitation bands in tropical and extratropical cyclones, and topographically generated weather systems such as mountain waves and sea and land breezes.” AMS Glossary of Meteorology, http://glossary.ametsoc.org

7 Latent and sensible heat fluxes are energy types that are exchanged (released from or absorbed) by a chemical substance or a thermodynamic system in the atmosphere. Latent heat flux is associated with changes of state or phase changes between liquids, gases, and solids without temperature change. Sensible heat is related solely to changes in temperature without modifications of the pressure or volume of the system. Latent heat flux modifies the atmospheric stability and sensible heat fluxes change the temperature of air masses and substances in the atmosphere.
